# Hyper-activated low-density neutrophil-derived NGAL predicts fatal SFTS: a multi-cohort and single-cell study

**DOI:** 10.1007/s15010-026-02840-9

**Published:** 2026-06-01

**Authors:** Guangqi Zhu, Zhuolin Han, Bei Jia, Yi Zhang, Yali Xiong, SuKun Kai, Jie Wang, Lifen Hu, Jihua Xue, Chao Wu, Jie Li, Wei Wu

**Affiliations:** 1 Department of Infectious Diseases, Nanjing Drum Tower Hospital, Affiliated Hospital of Medical School, Nanjing University, Nanjing, Jiangsu China; 2 Institute of Viruses and Infectious Diseases, Nanjing University, Nanjing, Jiangsu China; 3 State Key Laboratory for Diagnosis and Treatment of Infectious Diseases, National Clinical Research Center for Infectious Diseases, China-Singapore Belt and Road Joint Laboratory on Infection Research and Drug Development, National Medical Center for Infectious Diseases, Collaborative Innovation Center for Diagnosis and Treatment of Infectious Diseases, The First Affiliated Hospital, Zhejiang University School of Medicine, Hangzhou, 310003 Zhejiang China; 4 Department of Laboratory Medicine, The First Affiliated Hospital, Zhejiang University School of Medicine, Hangzhou, Zhejiang China; 5 Department of Infectious Diseases, The First Affiliated Hospital of Anhui Medical University, Hefei, Anhui China

**Keywords:** Severe fever with thrombocytopenia syndrome, Neutrophil gelatinase-associated lipocalin, Mortality, Biomarker

## Abstract

**Purpose:**

Severe fever with thrombocytopenia syndrome (SFTS) is an acute infectious disease with rapid progression and high mortality. This study aimed to assess the predictive power of serum neutrophil gelatinase-associated lipocalin (NGAL) for mortality risk and elucidate its cellular origin at the single-cell level.

**Methods:**

In this retrospective multicenter study, we analyzed clinical data from 215 SFTS patients across three Chinese hospitals (152 in the training cohort and 63 in the external validation cohort). We further analyzed publicly available single-cell RNA sequencing (scRNA-seq) data of peripheral blood mononuclear cells (PBMCs) from SFTS patients and healthy controls to investigate the cellular source of NGAL.

**Results:**

Serum NGAL was identified as an independent risk factor for 14-day and 28-day all-cause mortality (*P* < 0.05). High NGAL levels were linearly associated with increased mortality and remained stable across various clinical subgroups. Crucially, unbiased scRNA-seq analysis revealed that the elevated NGAL (encoded by the LCN2 gene) primarily originated from low-density neutrophils (LDNs) that co-purified with PBMCs. These LCN2-expressing LDNs were highly enriched in non-survivors and exhibited a hyper-inflammatory, interferon-stimulated transcriptomic signature.

**Conclusion:**

Our findings establish serum NGAL as a robust early predictor of mortality risk in SFTS patients, with its elevation intrinsically linked to the pathogenic emergence of highly activated LDNs, providing novel insights into the immunopathology of fatal SFTS.

**Supplementary Information:**

The online version contains supplementary material available at 10.1007/s15010-026-02840-9.

## Introduction

Severe fever with thrombocytopenia syndrome (SFTS) is an acute infectious disease caused by the dabie bandavirus (DBV). The clinical manifestations of this disease are characterized by acute onset, fever, and progressive thrombocytopenia and are often accompanied by leukopenia and multi-organ dysfunction [1]. A notable feature of this disease is its rapid onset and progression as well as its high case fatality rate, posing a serious threat to public health security [2]. Although progress has been made in recent years in the clinical understanding of SFTS, supportive treatment, and some antiviral strategies, the overall mortality rate remains high, especially among elderly patients and those with underlying comorbidities [3]. Developing and validating a non-invasive, cost-effective biomarker with high sensitivity and specificity is crucial for identifying patients at high risk of death in the early stages of the disease.

Despite increasing attention and research focus on identifying biomarkers capable of predicting mortality risk in patients with SFTS in recent years, the biomarkers reported in the existing literature still have many limitations. First, external validation is often lacking. For instance, Zhang et al. identified alkaline phosphatase as a risk factor using a retrospective multicenter cohort but did not validate their findings in an independent external cohort [4]. Second, the timing of biomarker measurement is not standardized across studies. Although some studies have performed dynamic monitoring, e.g., Hao et al., sampling time points vary considerably, hindering cross-study comparison [5]. Moreover, traditional serum inflammatory markers are not suitable for identifying patients at high risk of death in the early stages [6]. These shortcomings fail to fully meet clinical needs and underscore the pressing need for a robust, well-validated biomarker with a standardized sampling strategy to predict fatal outcomes in SFTS patients.

Neutrophil Gelatinase-Associated Lipocalin (NGAL), also known as Lipocalin-2 (LCN2), primarily functions in the transport of small ligands such as vitamins and pheromones. NGAL is produced not only by the kidneys but also by a variety of other cells and tissues, including neutrophils, lungs, stomach, colon, and heart [7]. NGAL has long been widely used in the diagnosis and monitoring of Acute Kidney Injury (AKI) [8]. In recent years, with further research into the functions of NGAL, increasing evidence has shown that NGAL has significant clinical application value in infectious diseases [9]. Elevated serum NGAL levels have been observed in patients with COVID-19 and dengue infections compared with healthy individuals [10, 11], suggesting that NGAL may have potential value in the diagnosis and prognostic assessment of infectious diseases. During severe systemic infections, hyper-inflammation triggers the massive activation and degranulation of neutrophils, frequently leading to the emergence of low-density neutrophils (LDNs). These LDNs co-purify with peripheral blood mononuclear cells (PBMCs) and are increasingly recognized as critical drivers of immune-mediated pathology and fatal outcomes [12]. However, whether NGAL can serve as a reliable prognostic marker for SFTS, and whether its elevation reflects this pathogenic neutrophil dysregulation, remains entirely unexplored.

Therefore, the present study aimed to investigate the feasibility and predictive efficacy of NGAL as a biomarker for mortality risk in a multicenter retrospective cohort study. We first conducted a multicenter retrospective cohort study to evaluate the predictive efficacy of serum NGAL for 14-day and 28-day mortality risks. Subsequently, to elucidate the immunopathological mechanisms driving NGAL elevation, we analyzed single-cell RNA sequencing (scRNA-seq) data of circulating PBMCs from SFTS patients. We hope this dual clinical-transcriptomic approach will not only validate NGAL’s potential for early risk assessment but also provide novel insights into the specific immune cell subsets driving fatal SFTS progression.

## Methods

### Key resources table

**Table:** 

Reagent or resource	Source	Identifier
*Biological samples*		
Human serum samples	Multicenter SFTS cohort	This paper
Human PBMC scRNA-seq data	GEO	GSE175499
*Critical commercial assays*		
Human NGAL ELISA kit	LiankeBio, Hangzhou, China	Cat# EK1116
*Deposited data*		
Single-cell RNA sequencing data	GEO	GSE175499
*Software and algorithms*		
R studio version v4.2.1	R software	https://www.r-project.org/

### Resource availability

#### Lead contact

Further information and requests for resources should be directed to and will be fulfilled by the lead contact, Wei Wu (zjuwuwei@zju.edu.cn).

### Materials availability

This study did not generate new unique reagents.

### Data and code availability

The single-cell RNA sequencing data used in this study are publicly available from the Gene Expression Omnibus (GEO) database under accession number GSE175499.

The clinical data used in this study are not publicly available due to privacy and ethical restrictions. De-identified data may be available from the corresponding authors upon reasonable request and with appropriate ethical approvals.

The original code for data analysis and statistical modeling is available from the corresponding authors upon reasonable request.

Any additional information required to reanalyze the data reported in this paper is available from the lead contact upon request.

### Experimental model and study participant details

#### Study design and participants

**Fig. 1:**
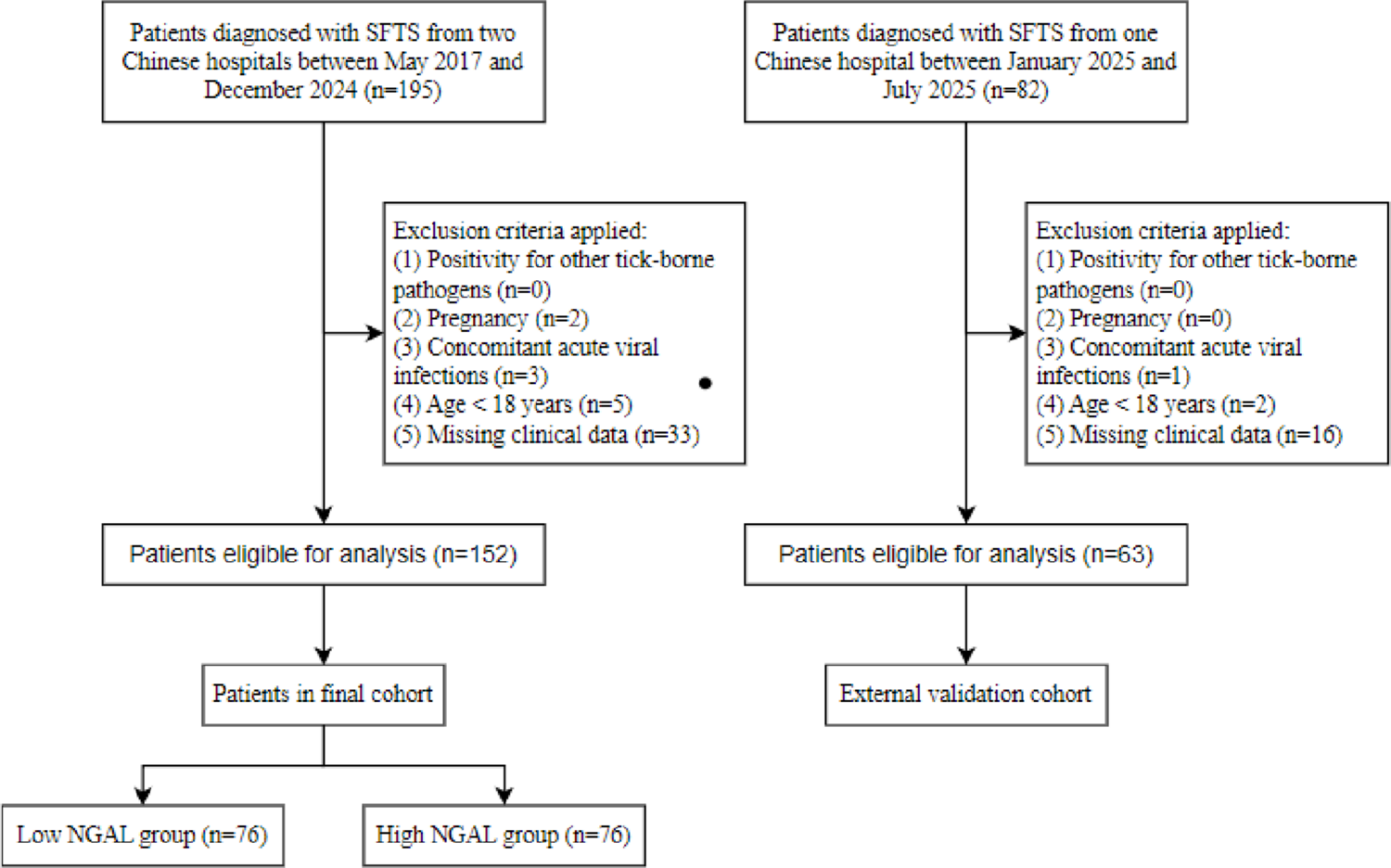
The flowchart of patient selection process

This retrospective multicenter study enrolled patients with SFTS from three centers: those admitted to Nanjing Drum Tower Hospital and the First Affiliated Hospital of Zhejiang University of Medicine between May 2017 and December 2024; and those admitted to the First Affiliated Hospital of Anhui Medical University between January 2025 and July 2025. The inclusion criteria were as follows: (1) diagnosis of SFTS and (2) age > 18 years. The exclusion criteria were as follows: (1) positivity for other tick-borne pathogens, (2) pregnancy, and (3) concomitant acute viral infections. A brief flowchart detailing the inclusion and exclusion process is provided (Fig. 1). The diagnosis of SFTS was based on the criteria in the “Guidelines for the Prevention and Control of Severe Fever with Thrombocytopenia Syndrome (2010 Edition)” issued by the former Ministry of Health of China [13]. All SFTS cases included in this study were confirmed by positive reverse transcription polymerase chain reaction (RT-PCR) detection.

### Ethics approval and consent to participate

The study was approved by the Ethics Committee of the First Affiliated Hospital, Zhejiang University School of Medicine (approval number: 2022 − 119); the Ethics Committee of Nanjing Drum Tower Hospital (approval number: 2022-238-02); and the Ethics Committee of Anhui Medical University (approval number: PJ 2024-07-99). Written informed consent was obtained from all the patients involved in this study. This study was conducted in accordance with the Declaration of Helsinki.

### Data collection

Eligible patients were invited to participate in this study. The study population comprised 152 SFTS patients from Nanjing Drum Tower Hospital and the First Affiliated Hospital of Zhejiang University of Medicine, while 63 SFTS patients from the First Affiliated Hospital of Anhui Medical University were used for external validation. Clinical data from these SFTS patients, including demographic information, clinical manifestations, laboratory tests, imaging examinations, and therapeutic regimens, were retrospectively collected from the hospital’s electronic medical record system using a standardized case report form. The covariates included age, sex, and comorbidities (hypertension, diabetes, and history of malignancy). The primary outcome was all-cause mortality.

### Measurement of serum NGAL

All serum samples were collected within the first 24 h of hospital admission, prior to the initiation of any specific antiviral or immunosuppressive therapy, to establish the baseline NGAL level for each patient. Serum NGAL levels were measured using a commercial human NGAL enzyme-linked immunosorbent assay (ELISA) kit (Cat. No. EK1116; LiankeBio, Hangzhou, China), according to the manufacturer’s instructions.

### Acquisition and processing of single-cell RNA sequencing (scRNA-seq) data

To explore the cellular correlates of circulating NGAL, we obtained scRNA-seq data of circulating peripheral blood mononuclear cells (PBMCs) from SFTS patients and healthy individuals from a previously published dataset (GSE175499) that uniquely captured Low-Density Neutrophils (LDNs) to evaluate LCN2 gene transcription. The Cell Ranger toolkit (V3.1.0) was applied to align reads and generate the gene-cell matrix against the reference genome GRCh38. The expression profile matrix was then loaded into Seurat (version 4.1.0) for quality control, filtering, and clustering. For each cell, we retained high-quality cells with 500–8000 detected genes and no more than 25% mitochondrial gene counts. Cells were projected into 2D space using Uniform Manifold Approximation and Projection (UMAP).

### Cell type annotation and identification of low-density neutrophils (LDNs)

The Seurat FindAllMarkers function was employed to identify marker genes for each cell cluster. Clusters were initially annotated using canonical markers for specific immune lineages. Notably, while standard density gradient centrifugation typically depletes normal-density neutrophils, severe inflammatory states induce the release of low-density neutrophils (LDNs) that co-segregate with the mononuclear cell fraction. Therefore, cells expressing canonical neutrophil markers (e.g., CSF3R, FCGR3B, CXCR2) within the PBMC dataset were deliberately retained and annotated as LDNs. To ensure the purity of this LDN population, we performed sub-clustering on the initially identified clusters and excluded potential doublets or contaminating cells expressing markers for other lineages (e.g., CD3 and IL7R for T cells, GNLY for NK cells). The remaining high-quality LDNs underwent a second round of standard preprocessing and sub-clustering to explore intra-population heterogeneity.

### Differentially expressed genes (DEGs) and functional pathway enrichment

Differentially expressed genes (DEGs) between survivors and non-survivors within the LDN population were identified using the FindMarkers function in Seurat (logfc.threshold = 0.25). Functional enrichment analysis of the identified gene sets was performed based on the Kyoto Encyclopedia of Genes and Genomes (KEGG) pathway and Gene Ontology (GO) databases using the clusterProfiler package. Gene Set Variation Analysis (GSVA) was used to assess the performance of gene sets for pathway enrichment at the sample level.

### Statistical analysis

R 4.2.1 software was used to process and analyze the data. Based on the optimal cutoff value of NGAL for predicting 28-day all-cause mortality in the cohort, the patients were divided into two groups: Low NGAL (NGAL < 60.597 pg/ml) and High NGAL (NGAL ≥ 60.597 pg/ml) (Table S2). Continuous variables with normal distribution and homogeneity of variance were represented by mean ± standard deviation (x ± s), whereas non-normal distribution and heterogeneity of variance are represented by median (IQR). Categorical variables are described using frequencies and percentages to characterize the baseline features of the patients. Univariate and multivariate Cox regression analyses were conducted to calculate hazard ratios (HRs) and 95% confidence intervals (95% CI) for all-cause mortality. Kaplan-Meier curves were used to compare survival times among patients with different NGAL levels. Restricted cubic spline (RCS) curve fitting was used to assess the linear relationship between NGAL levels and all-cause mortality. Receiver operating characteristic (ROC) curve analysis was used to compare the predictive efficacy of NGAL with that of other independent risk factors. *P* < 0.05 is considered statistically significant. For scRNA-seq data, enriched pathways and DEGs were identified based on adjusted $P$ < 0.05, with the false discovery rate estimated using the Benjamini-Hochberg method. A $P$ < 0.05 was considered statistically significant across all analyses.

## Results

### Baseline characteristics of patients with SFTS

A total of 152 patients were included in the study population, comprising 136 survivors and 16 non-survivors. Comparison of baseline characteristics between survivors and non-survivors revealed that the patients’ median age was 63.0 years, and 42.8% were male. The days from symptom onset to admission were 6.7 ± 3.1 days, and the hospitalization days were 11.1 ± 8.1 days. Patients in the non-survivor group were significantly older, had a higher proportion of complications, and exhibited elevated serum NGAL levels (*P* < 0.05). Regarding organ involvement, compared with survivors, non-survivors had significantly higher proportions of organ failure, a greater number of damaged organs, and a greater number of failed organs (*P* < 0.001) (Table 1). However, no statistical differences were observed between the two groups in terms of sex or history of comorbidities (*P* > 0.05). Further comparison between the high and low NGAL groups showed that patients with high NGAL levels had a higher incidence of organ failure and 28-day all-cause mortality (*P* < 0.05) (Table S2).

**Table 1:** Baseline characteristics between survivors and non-survivors

Characteristics	Total (*n* = 152)	Survivors (*n* = 136)	Non-survivors (*n* = 16)	*p*
Age, Mean ± SD	63.0 (54.0, 71.2)	62.0 (54.0, 71.0)	73.5 (65.8, 77.5)	0.003
Gender, n (%)				0.536
Male	65 (42.8)	57 (41.9)	8 (50)	
Female	87 (57.2)	79 (58.1)	8 (50)	
Days from symptom onset to admission, Mean ± SD	6.7 ± 3.1	6.7 ± 3.2	6.8 ± 2.6	0.925
Hospitalization days, Mean ± SD	11.1 ± 8.1	11.7 ± 8.2	6.1 ± 4.5	0.008
Clinical typing, n (%)				< 0.001
1	13 (8.6)	13 (9.6)	0 (0)	
2	69 (45.4)	69 (50.7)	0 (0)	
3	41 (27.0)	39 (28.7)	2 (12.5)	
4	29 (19.1)	15 (11)	14 (87.5)	
Comorbidity				
Number of comorbidities, n (%)				0.36
0	67 (44.1)	61 (44.9)	6 (37.5)	
1	32 (21.1)	28 (20.6)	4 (25)	
2	29 (19.1)	26 (19.1)	3 (18.8)	
3	13 (8.6)	12 (8.8)	1 (6.2)	
4	7 (4.6)	6 (4.4)	1 (6.2)	
5	3 (2.0)	3 (2.2)	0 (0)	
6	1 (0.7)	0 (0)	1 (6.2)	
Hypertension, n (%)	45 (29.6)	39 (28.7)	6 (37.5)	0.563
Diabetes, n (%)	20 (13.2)	18 (13.2)	2 (12.5)	1
Cancer, n (%)	9 (5.9)	7 (5.1)	2 (12.5)	0.241
Clinical features				
Fever, n (%)				0.518
37.3 ~ 38	23 (15.1)	19 (14)	4 (25)	
38.1 ~ 39	59 (38.8)	54 (39.7)	5 (31.2)	
39.1 ~ 41	70 (46.1)	63 (46.3)	7 (43.8)	
Digestive symptoms, n (%)	81 (53.3)	74 (54.4)	7 (43.8)	0.419
Sickness, n (%)	30 (19.7)	24 (17.6)	6 (37.5)	0.09
Disturbance of consciousness, n (%)	43 (28.3)	27 (19.9)	16 (100)	< 0.001
Laboratory tests				
NGAL(pg /ml), median (IQR)	50.4 (27.0, 77.9)	47.9 (25.7, 75.0)	78.0 (58.8, 104.2)	0.001
<60.597, n (%)	95 (62.5)	91 (66.9)	4 (25)	0.001
≥ 60.597, n (%)	57 (37.5)	45 (33.1)	12 (75)	
White blood cell (10^9^/L), median (IQR)	3.0 (1.9, 5.1)	3.0 (2.0, 5.0)	3.0 (1.9, 5.8)	0.974
Red blood cell(10^12^/L), median (IQR)	4.3 (4.0, 4.7)	4.3 (4.0, 4.7)	4.0 (3.8, 4.5)	0.328
Platelet (10^9^/L), median (IQR)	65.0 (41.0, 91.5)	67.0 (42.0, 93.2)	52.0 (28.8, 70.8)	0.047
Neutrophils (%), median (IQR)	64.8 (50.9, 76.6)	64.6 (50.3, 76.4)	65.7 (53.2, 78.3)	0.435
Lymphocytes (%), median (IQR)	26.1 (16.4, 38.1)	26.1 (16.7, 38.1)	26.8 (9.8, 34.9)	0.469
Hemoglobin (g/L), median (IQR)	129.0 (117.0, 142.0)	130.0 (117.0, 142.0)	127.0 (110.5, 139.8)	0.642
Alanine transaminase (U/L), median (IQR)	63.0 (38.8, 117.0)	62.5 (37.6, 116.2)	100.0 (49.0, 182.0)	0.085
Lactate dehydrogenase (U/L), median (IQR)	649.0 (316.8, 1057.2)	585.5 (303.0, 1000.0)	1819.5 (615.0, 4474.2)	0.002
Total bilirubin (µmol/L), median (IQR)	9.4 (6.2, 13.7)	9.4 (6.4, 13.6)	9.9 (5.7, 16.1)	0.773
Direct bilirubin (µmol/L), median (IQR)	3.7 (2.3, 6.1)	3.6 (2.2, 5.7)	3.9 (2.5, 12.7)	0.184
Creatine kinase (U/L), median (IQR)	219.5 (92.0, 693.5)	203.5 (87.0, 649.8)	592.5 (193.0, 1432.8)	0.063
Brain natriuretic peptide (pg/ml), median (IQR)	7.1 (2.5, 50.6)	7.1 (2.4, 50.6)	12.4 (3.5, 90.2)	0.24
Triglycerides (mmol/L), median (IQR)	1.7 (0.9, 2.6)	1.7 (1.0, 2.6)	1.0 (0.6, 2.5)	0.118
Total cholesterol (mmol/L), median (IQR)	2.9 (1.7, 3.4)	3.0 (1.8, 3.4)	2.0 (0.4, 3.3)	0.045
High-density lipoprotein cholesterol (mmol/L), median (IQR)	1.5 (0.9, 191.2)	1.2 (0.9, 147.4)	254.0 (1.1, 475.4)	0.025
Low-density lipoprotein cholesterol (mmol/L), median (IQR)	0.7 (0.0, 1.5)	0.6 (0.0, 1.5)	0.8 (0.2, 1.3)	0.458
Globulin (g/L), median (IQR)	28.3 (24.0, 32.9)	27.8 (23.9, 32.4)	31.8 (25.7, 37.6)	0.049
Creatinine (µmol/L), median (IQR)	69.3 (55.5, 90.8)	67.0 (52.8, 86.2)	96.5 (79.7, 131.5)	< 0.001
Urine protein, n (%)				0.09
0	27 (17.8)	27 (19.9)	0 (0)	
1	33 (21.7)	27 (19.9)	6 (37.5)	
2	49 (32.2)	45 (33.1)	4 (25)	
3	39 (25.7)	34 (25)	5 (31.2)	
4	4 (2.6)	3 (2.2)	1 (6.2)	
Blood urea nitrogen (mg/dL), median (IQR)	5.2 (3.5, 8.2)	5.1 (3.4, 7.6)	7.5 (4.9, 10.8)	0.038
C-reactive protein (mg/L), median (IQR)	4.9 (2.8, 9.3)	4.4 (2.5, 8.0)	9.1 (5.8, 24.2)	0.007
Prothrombin time (s), median (IQR)	11.6 (10.8, 12.3)	11.5 (10.8, 12.2)	11.9 (11.5, 12.4)	0.16
International normalized ratio, median (IQR)	1.0 (0.9, 1.1)	1.0 (0.9, 1.1)	1.0 (1.0, 1.1)	0.084
Amylase (U/L), median (IQR)	105.0 (69.0, 168.0)	104.0 (69.0, 162.8)	139.1 (87.0, 291.5)	0.051
Lipase (U/L), median (IQR)	141.5 (36.0, 353.0)	133.0 (36.0, 260.0)	585.0 (87.8, 1615.0)	0.028
Treatment				
Corticosteroid Therapy, n (%)	41 (27.0)	31 (22.8)	10 (62.5)	0.002
Ribavirin treatment, n (%)	140 (92.1)	125 (91.9)	15 (93.8)	1
Gamma globulin therapy, n (%)	58 (38.2)	48 (35.3)	10 (62.5)	0.034
Complication				
Myocarditis, n (%)	28 (18.4)	20 (14.7)	8 (50)	0.002
Encephalitis, n (%)	34 (22.4)	23 (16.9)	11 (68.8)	< 0.001
Pulmonary infection, n (%)	49 (32.2)	39 (28.7)	10 (62.5)	0.006
Organ failure, n (%)	21 (13.8)	9 (6.6)	12 (75)	< 0.001
The number of damaged organs, n (%)				< 0.001
0	46 (30.3)	45 (33.1)	1 (6.2)	
1	23 (15.1)	23 (16.9)	0 (0)	
2	25 (16.4)	25 (18.4)	0 (0)	
3	30 (19.7)	26 (19.1)	4 (25)	
4	17 (11.2)	10 (7.4)	7 (43.8)	
5	7 (4.6)	5 (3.7)	2 (12.5)	
6	2 (1.3)	1 (0.7)	1 (6.2)	
7	2 (1.3)	1 (0.7)	1 (6.2)	
The number of failed organs, n (%)				< 0.001
0	131 (86.2)	127 (93.4)	4 (25)	
1	14 (9.2)	8 (5.9)	6 (37.5)	
2	4 (2.6)	1 (0.7)	3 (18.8)	
3	2 (1.3)	0 (0)	2 (12.5)	
4	1 (0.7)	0 (0)	1 (6.2)	

*IQR *Interquartile range, *SD* Standard deviation

### The relationship between NGAL and all-cause mortality

**Fig. 2:**
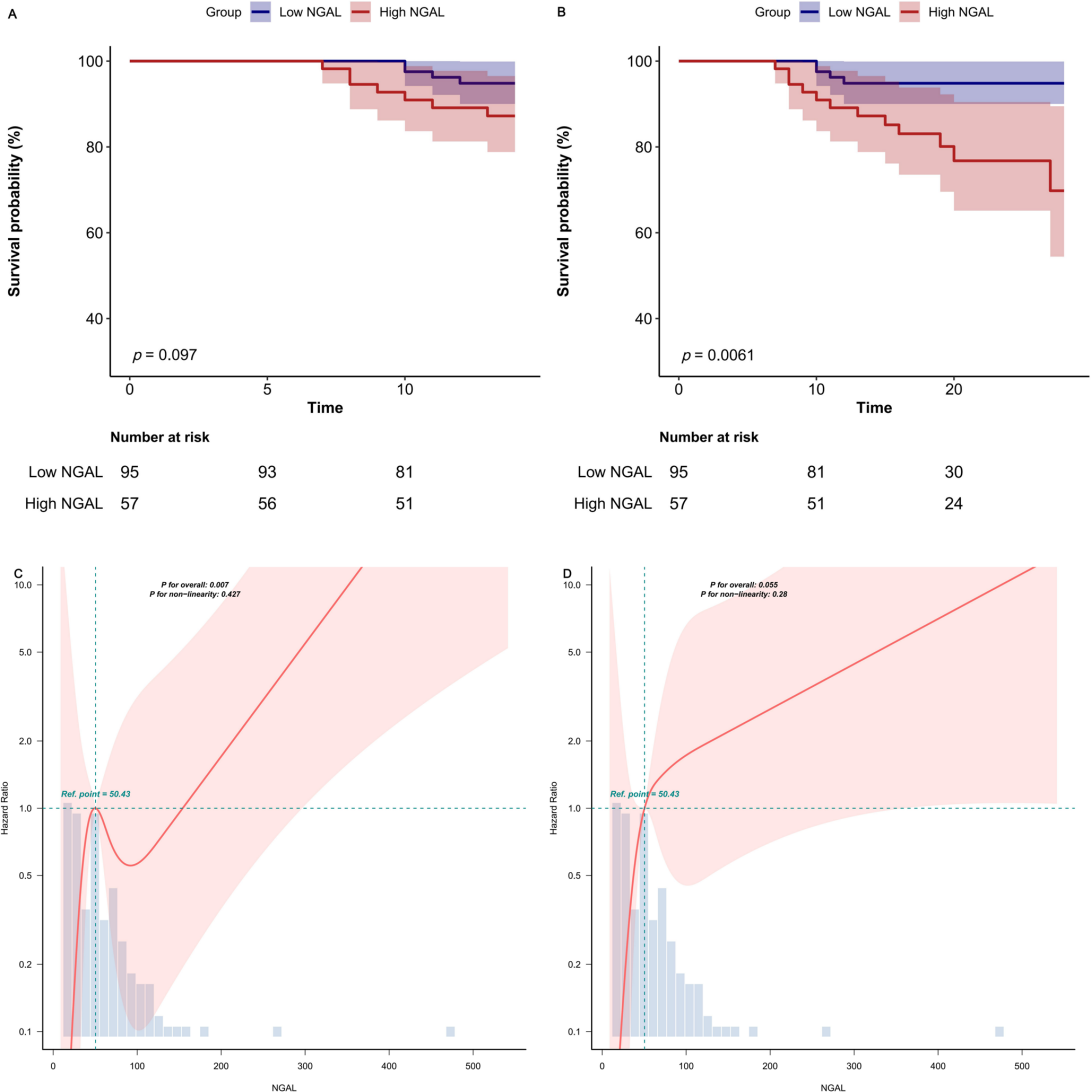
Association of NGAL with mortality in SFTS patients. **A** Kaplan-Meier estimation curves for 14-day all-cause mortality according to NGAL groups. **B** Kaplan-Meier estimation curves for 28-day all-cause mortality according to NGAL groups. **C** Restricted cubic spline analyses of NGAL as a continuous variable for 14-day all-cause mortality. **D** Restricted cubic spline analyses of NGAL as a continuous variable for 28-day all-cause mortality. Adjusted for age, gender, and underlying diseases (hypertension, diabetes, cancer). The shaded red areas indicate the 95% confidence intervals

Using univariate and multivariate Cox regression analyses, we found that NGAL as a continuous variable was an independent risk factor for 14-day all-cause mortality (HR: 1.01; 95% CI: 1.005–1.016; *P* < 0.001) and 28-day all-cause mortality (HR: 1.007; 95% CI: 1.002–1.011; *P* < 0.05) in patients with SFTS. When NGAL was analyzed as a binary variable, it was an independent risk factor for 28-day all-cause mortality (HR: 4.816; 95% CI: 1.468–15.793; *P* < 0.05) (Table 2). Compared with the low NGAL group, patients in the high NGAL group exhibited a significantly lower 28-day survival rate (*P* < 0.05) (Fig. 2A and B). Furthermore, NGAL levels showed a positive linear association with 14-day all-cause mortality (*P* < 0.05) (Fig. 2C and D). Subgroup analyses revealed that the association between NGAL levels and 28-day all-cause mortality remained consistent across all evaluated subgroups (Figure S1). Sensitivity analyses demonstrated that after excluding patients with severe renal dysfunction (Table S3), as well as those with hypertension (Table S4), the associations between NGAL and all-cause mortality remained consistent with the overall cohort, supporting the robustness of our findings. Additionally, we performed a sensitivity analysis further adjusting for clinical typing and treatment strategies (corticosteroid, ribavirin, and gamma globulin therapy). NGAL remained a significant independent predictor of 28-day mortality in this fully adjusted model (Table S5). Furthermore, after adjusting for all recorded complications, NGAL remained significantly associated with 28-day mortality (Table S6).

**Table 2:** Cox proportional hazards models analyses for NGAL and mortality outcomes in SFTS patients

	Univariate model		Multivariate model l		Multivariate model 2	
	HR (95%CI)	*P*	HR (95%CI)	*P*	HR (95%CI)	*P*
14-day all-cause mortality						
NGAL	1.007 (1.003 ~ 1.011)	0.001	1.01 (1.004 ~ 1.015)	< 0.001	1.01 (1.005 ~ 1.016)	< 0.001
Low NGAL	Reference		Reference		Reference	
High NGAL	2.707 (0.792 ~ 9.251)	0.112	2.544 (0.742 ~ 8.72)	0.137	1.829 (0.503 ~ 6.645)	0.359
28-day all-cause mortality						
NGAL	1.005 (1.002 ~ 1.009)	0.006	1.006 (1.002 ~ 1.01)	0.005	1.007 (1.002 ~ 1.011)	0.002
Low NGAL	Reference		Reference		Reference	
High NGAL	4.284 (1.378 ~ 13.317)	0.012	4.148 (1.335 ~ 12.89)	0.014	4.816 (1.468 ~ 15.793)	0.01

*HR* hazard ratio, *CI* confidence interval

Multivariate model 1: age, gender were adjusted

Multivariate model 2: age, gender, comorbidity (hypertension, diabetes, cancer) were adjusted

### The predictive efficacy of NGAL for all-cause mortality outcomes

**Fig. 3:**
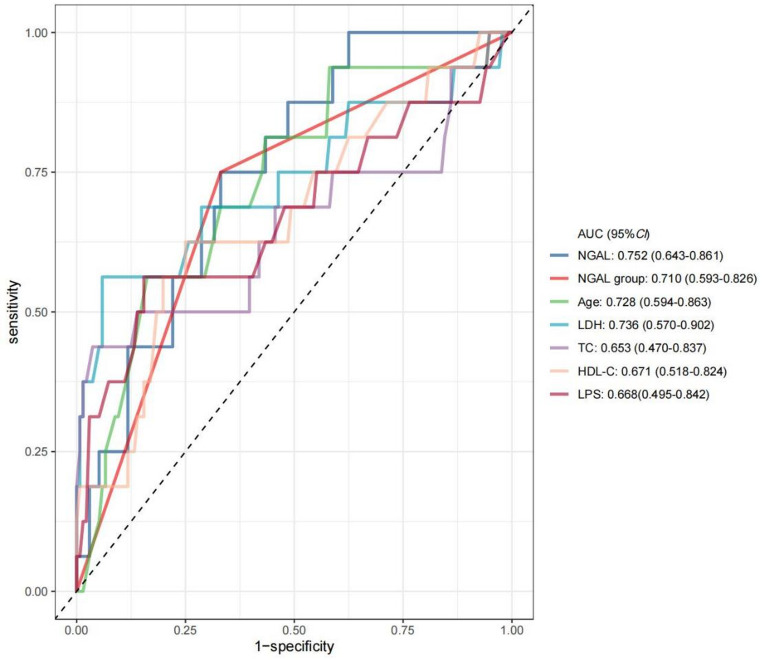
ROC curve of NGAL compared with other risk factors for 28-day all-cause mortality in SFTS patients. ROC: receiver operating characteristic

The optimal cutoff values of serum NGAL for predicting 14-day and 28-day all-cause mortality were determined to be 33.915 pg/ml and 60.597 pg/ml, respectively (Table S1). NGAL showed a moderate discriminative ability, with an AUC of 0.752 for predicting 28-day all-cause mortality (Figure S2). Stepwise regression analysis identified age, NGAL, LDH, TC, HDL-C, and LPS as independent risk factors for 28-day mortality (Table S7). Notably, NGAL showed a moderate predictive performance, with an AUC of 0.752, which was numerically higher than that of some other indicators (Fig. 3). Importantly, in an independent external validation cohort, NGAL maintained robust predictive power for 28-day mortality, consistently outperforming the other identified risk factors (Figure S3).

### Low-density neutrophils (LDNs) are enriched in fatal SFTS and exhibit a distinct LCN2-driven transcriptomic profile

**Fig. 4:**
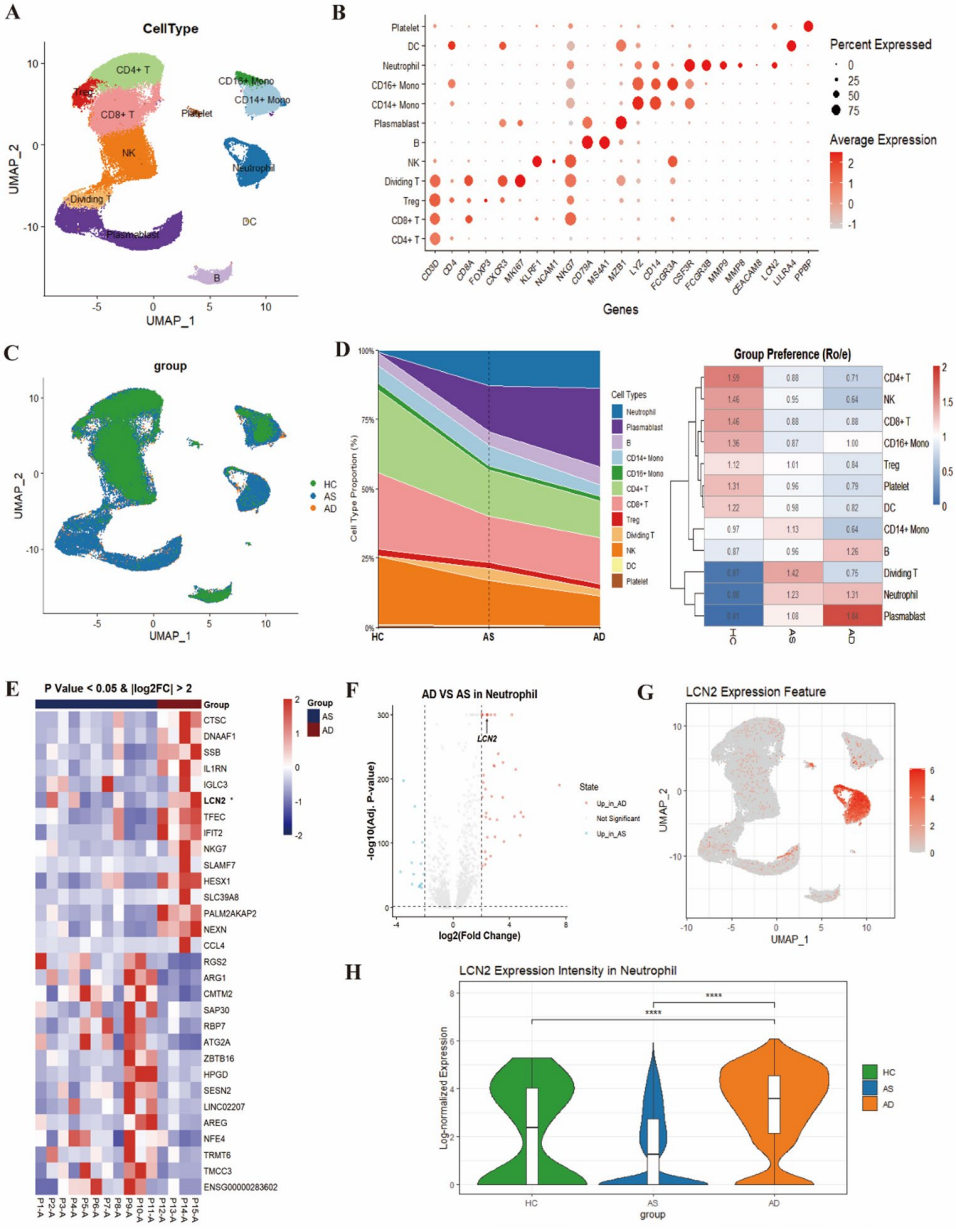
LDNs are enriched in fatal SFTS and exhibit a distinct LCN2-driven transcriptomic profile. LCN2: lipocalin-2 (gene encoding NGAL); UMAP: uniform manifold approximation and projection; DEGs: differentially expressed genes; HC: healthy control; AS: acute survived; AD: acute deceased. **A** UMAP plot of immune cell type identified by scRNA-seq. **B** Dotplot displaying representative marker genes defining each cell type. **C** UMAP plot showing the distribution of immune cells across the Healthy Control (HC), Acute Survived (AS), and Acute Deceased (AD) groups. **D** Stacked bar plot and heatmap depicting the variation in cell proportions among the three groups. **E** Heatmap of DEGs in LDNs from non-survivors versus survivors(*P* < 0.05 & |log2FC| > 2). **F** Volcano plot of DEGs in LDNs from deceased patients versus survivors, with LCN2 labeled. **G** Visualization of LCN2 expression features in the UMAP plot. **H** Violin plot comparing LCN2 expression in LDNs among the three groups

Given the robust prognostic value of serum NGAL, we next sought to determine its possible cellular correlates and immunopathological role by analyzing single-cell RNA sequencing (scRNA-seq) profiles of circulating immune cells. From previously published datasets, we obtained circulating PBMC samples from 4 healthy controls, 11 survivors, and 4 non-survivors, yielding 95,739 cells for analysis. UMAP dimensionality reduction and clustering revealed main immune cell populations, including T cells, B cells, NK cells, and monocytes (Fig. 4A and B). Notably, while standard density gradient centrifugation typically depletes normal-density neutrophils, we identified a substantial neutrophil population co-purifying with the PBMC fraction. These cells represent low-density neutrophils (LDNs), which are widely recognized as a hallmark of severe systemic inflammation. We observed that the proportions of these LDNs, along with B cells and plasmablasts, were higher in SFTS patients compared with healthy controls, and were further enriched in non-survivors (Fig. 4C and D).

To explore the molecular mechanisms driving LDN-mediated pathology in fatal SFTS, we performed an unbiased differential gene expression analysis comparing LDNs from Acute Deceased (AD) and Acute Survived (AS) patients. As illustrated in the heatmap (Fig. 4E) and volcano plot (Fig. 4F), a distinct set of genes was profoundly altered in the LDNs of non-survivors (*P* < 0.05). Notably, LCN2 (the gene encoding NGAL) was identified as one of the upregulated genes in the non-survivors. Visualization of the LCN2 expression patterns revealed that its transcripts were highly enriched within the LDN population (Fig. 4G). Further analysis indicated that the LCN2 expression level in LDNs was higher in non-survivors compared to survivors (Fig. 4H).

### LCN2 is highly expressed in a specific LDN subset characterized by hyper-inflammation and interferon responses

**Fig. 5:**
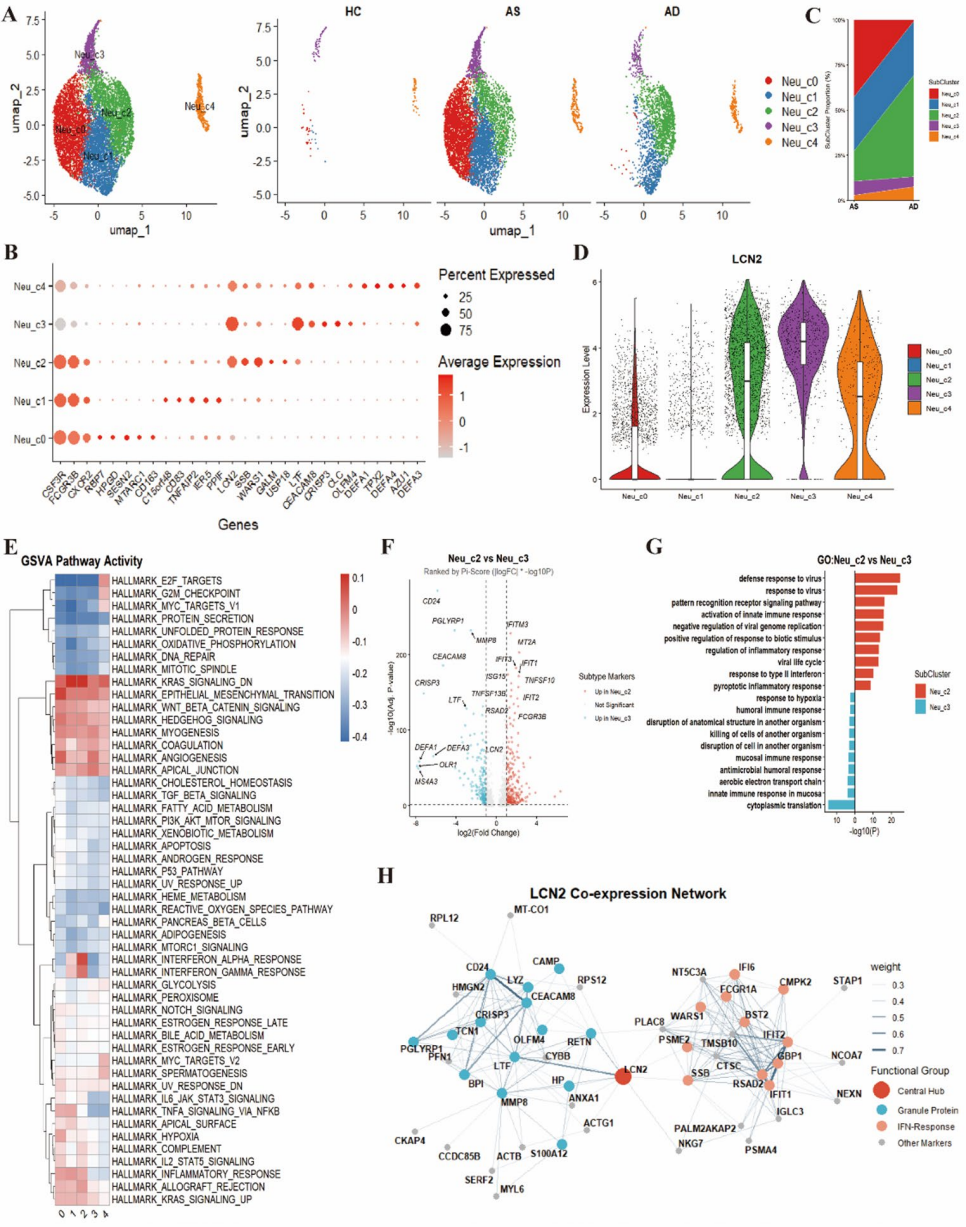
LCN2 is highly expressed in a specific LDN subset characterized by hyper-inflammation and interferon responses. GSVA: Gene Set Variation Analysis; GO: Gene Ontology; ISGs: interferon-stimulated genes. **A** UMAP plot illustrating LDN subsets (Neu_c0-Neu_c4) (left) and the cell distribution among the three groups (HC, AS, and AD) (right). **B** Dot plot displaying the expression of CSF3R, FCGR3B, CXCR2, and the top 5 marker genes of the five subsets. **C** Stacked bar plot displaying the proportion distribution of each LDN subset in survived and deceased patients. **D** Violin plot with box plot showing the expression level of LCN2 in each LDN subset. **E** GSVA pathway plot of each LDN subset. **F** Volcano plot of differentially expressed genes (DEGs) between Neu_c2 and Neu_c3. **G** GO analysis of DEGs between Neu_c2 and Neu_c3. **H** LCN2 co-expression network

To resolve the heterogeneity of these pathogenic LDNs, a total of 9,190 LDNs were extracted and re-clustered into five distinct subsets (Neu_c0-Neu_c4) (Fig. 5A). A dot plot visualized the expression patterns of mature neutrophil markers (CSF3R, FCGR3B, CXCR2) alongside the top 5 differentially expressed genes of each subset (Fig. 5B). We found that LCN2 was the most prominently expressed gene in the Neu_c2 subset (Fig. 5B and D). By comparing cellular proportions, we discovered that this Neu_c2 subset was significantly enriched in non-survivors, indicating its connection with fatal outcomes (Fig. 5C).

GSVA pathway analysis demonstrated that the Neu_c2 subset specifically activated interferon $\alpha$ and $\gamma$ responses (Fig. 5E). To further investigate its molecular signature, we compared Neu_c2 with the Neu_c3 subset (which mainly expresses granule proteins like LTF, MMP8, and CEACAM8). Volcano plot analysis revealed that Neu_c2 is defined by a significant upregulation of interferon-stimulated genes (ISGs), including IFITM3, IFIT1, IFIT3, and ISG15 (Fig. 5F). GO enrichment analysis corroborated the significant enrichment of pathways such as “defense response to virus,” “regulation of inflammatory response,” and “response to type II interferon” in the Neu_c2 subset (Fig. 5G). Finally, a co-expression network constructed from all LDNs with detectable LCN2 expression revealed that LCN2 acts as a central hub, showing strong co-expression with multiple interferon response genes and granule proteins (Fig. 5H). Collectively, these single-cell transcriptomic findings suggest that the clinical elevation of serum NGAL maybe associated with the enrichment of a hyper-inflammatory, LCN2-expressing LDN subset during fatal SFTS.

## Discussion

In this study, we comprehensively investigated the value of NGAL as a novel biological marker for predicting mortality risk in patients with SFTS. Our findings revealed that NGAL was associated with adverse outcomes in patients with SFTS and serves as an independent risk factor for 28-day mortality. Notably, NGAL exhibited a moderate and competitive predictive performance compared with these indicators in our cohort.

Our study found a significant difference in NGAL levels between the mortality and survivors of SFTS patients. Consistent with our findings, previous studies have shown that NGAL levels are elevated in various infectious diseases and that higher NGAL levels are associated with disease severity and adverse outcomes [14, 15]. This correlation may be attributed to the upregulation of NGAL expression in neutrophils, epithelial cells, or hepatocytes induced by inflammatory cytokines in infectious diseases [16]. Our single-cell transcriptomic analysis extends these findings by revealing a strong inferred transcriptomic association with this profound elevation in SFTS. Specifically, we observe that robust LCN2 expression is highly enriched within a low-density neutrophil (LDN) subset emerging in the peripheral blood. The Neu_c2 LDN subset, characterized by intense interferon α and γ responses, shows significant enrichment in non-survivors, suggesting that the elevated LCN2 transcript levels reflect an underlying state of hyper-inflammatory neutrophil dysregulation. While these findings do not establish causality between the LCN2 transcription observed in LDNs and the actual secretion of NGAL protein into the circulation, this biological context supports the rationale for using NGAL as a prognostic marker and highlights LDNs and their inflammatory pathways as potential areas of interest for future therapeutic investigation in severe SFTS.

A high NGAL level (≥ 60.597 pg/ml) was an independent risk factor for 28-day all-cause mortality. Notably, the same NGAL cutoff value was not an independent risk factor for 14-day all-cause mortality. Similarly, no significant difference was observed in the 14-day survival rates between patients with different NGAL levels, whereas patients with high NGAL levels had lower 28-day survival rates than those with low NGAL levels. These findings suggest that NGAL is a valuable biomarker for assessing the risk of death in late-stage SFTS. We also found that when NGAL was treated as a continuous variable, it had a linear relationship with 14-day all-cause mortality, indicating that an increase in NGAL levels was associated with an increase in 14-day all-cause mortality. Although an increasing trend was observed in the relationship between NGAL levels and 28-day all-cause mortality, no linear relationship was observed. This suggests that NGAL has quantitative value in the early warning of death risk in patients with SFTS. The results of the Cox regression analysis, survival analysis, and restricted cubic spline (RCS) curve fitting for NGAL were not consistent with respect to the assessment of 14-day all-cause mortality. This may be because NGAL is not only expressed in neutrophils, but also in tubular cells of the kidney, cardiomyocytes, pulmonary epithelial cells, hepatocytes, gastric mucosal cells, colonic epithelial cells, and macrophages [17]. In the early stages of the disease, the mechanisms leading to rapid death in SFTS patients, such as hemophagocytic lymphohistiocytosis and viral encephalitis, may not be entirely dependent on NGAL as a biomarker [2, 18]. However, as the disease progresses, the profound expansion of the LCN2-expressing LDN subset reflects an overwhelming systemic inflammatory response. The excessive interferon-stimulated gene (ISG) activation within these LDNs likely contributes directly to late-stage immune-mediated tissue damage and the observed higher 28-day mortality. In summary, NGAL as a continuous variable has more practical value in early risk warnings. If the focus is on mid-to-late-stage death risk, NGAL should be prioritized as a binary variable for risk stratification in the models.

The mortality risk in patients with SFTS typically varies significantly across different ages, sexes, severities of clinical manifestations, and treatment modalities [19, 20]. To address this, we conducted further subgroup analyses, which yielded consistent results that were not influenced by factors such as patient age, sex, severity of clinical manifestations, or treatment strategies. This indicates robust reliability for mortality risk prediction in patients with SFTS. Furthermore, given the close association between NGAL and renal function in clinical practice, where NGAL is often used as a biomarker for kidney injury [21], we excluded patients with serum creatinine (Cr) levels > 177 µmol/L (indicative of decompensated chronic renal insufficiency, renal failure, and uremia) and performed a multivariate Cox regression analysis. These results remained consistent, further supporting the independent value of NGAL in predicting mortality risk. Some studies have found that patients with hypertension have higher serum NGAL levels than individuals with normal blood pressure. In secondary hypertension caused by primary aldosteronism, dendritic cells and macrophages are stimulated during periods of aldosterone excess, leading to increased NGAL synthesis, further promoting vascular fibrosis and the development of hypertension [22, 23]. However, in our study, we found that in patients without hypertension, high NGAL levels were closely related to 14-day and 28-day all-cause mortality. In contrast, NGAL level was not a risk factor for all-cause mortality in patients with hypertension. This may reflect baseline elevation of serum NGAL in hypertensive patients, and further elevation of NGAL after DBV infection may not effectively reflect changes in mortality risk.

Previous studies on biomarkers for mortality risk in SFTS have shown that although these indicators possess certain predictive values, their conclusions are often inconsistent or based on small sample sizes [24]. Recently, LDH has been proposed that LDH is a potential biomarker for predicting mortality risk in patients with SFTS [25]. In the present study, we identified independent risk factors associated with 28-day mortality and performed ROC analysis. The results showed that the AUC for NGAL (0.752) was numerically higher than that for LDH and other identified independent risk factors, without claiming statistical superiority. This indicates that NGAL has a higher sensitivity and specificity in predicting 28-day all-cause mortality, and its predictive efficacy is superior to that of LDH and other independent risk factors. This finding further confirms the importance of NGAL level as a predictive indicator for assessing patient mortality risk.

This study has some limitations. First, this was a retrospective study, which is inherently subject to selection and information bias. Second, the sample size in this study was relatively small. In addition, the dynamic changes in NGAL levels were not explored in this study. While our single-cell analysis provided valuable transcriptomic insights, the non-survivors in this dataset contained only 4 patients, which severely limited the strength and applicability of the results. Future studies with larger, prospectively collected single-cell cohorts are required. Additionally, due to the lack of experimental validation, the single-cell results should be interpreted as exploratory, as the detection of LCN2 mRNA in LDNs only represented an associative finding and did not provide direct evidence of actual NGAL protein secretion into the circulation. To definitively verify the cellular origin of serum NGAL, further wet-lab validation using patient-derived samples is necessary. Specifically, such validation requires demonstrating LCN2 transcription via RT-qPCR in isolated neutrophils, and providing evidence of NGAL protein secretion via ELISA on supernatants or intracellular flow cytometry, ideally accompanied by a correlation with serum levels. Furthermore, the direct functional impact of these LCN2-high LDNs on end-organ injury in SFTS requires further experimental investigation. Further validation in larger-scale, multicenter, longitudinal cohorts is needed in the future.

## Conclusion

In summary, NGAL is closely associated with mortality risk in patients with SFTS and demonstrates good predictive efficacy for both 14-day and 28-day all-cause mortalities. This prognostic value is mechanistically supported by the robust emergence of LCN2-expressing low-density neutrophils, marking a state of severe immune dysregulation. Therefore, NGAL levels should be closely monitored during hospitalization of patients with SFTS. Monitoring and evaluating NGAL levels can help physicians to identify patients at a higher risk of death at an early stage and guide intensified supportive care to improve outcomes.

## Supplementary Information

Below is the link to the electronic supplementary material.

Supplementary Material 1

## Data Availability

The single-cell RNA sequencing data used in this study are publicly available from the Gene Expression Omnibus (GEO) database under accession number GSE175499.The clinical data used in this study are not publicly available due to privacy and ethical restrictions. De-identified data may be available from the corresponding authors upon reasonable request and with appropriate ethical approvals.The original code for data analysis and statistical modeling is available from the corresponding authors upon reasonable request.Any additional information required to reanalyze the data reported in this paper is available from the lead contact upon request.
