# Supplementary material for: Hyper-activated low-density neutrophil-derived NGAL predicts fatal SFTS: a multi-cohort and single-cell study
Source: Infection. 2026 Jun 1;54(4):2085–97. doi: 10.1007/s15010-026-02840-9 (PMC13476323; doi:10.1007/s15010-026-02840-9)
Supplement: Supplementary file 1 [file 15010_2026_2840_MOESM1_ESM.docx]

**Items**

**Figure S1.** Subgroup analysis of associations between NGAL and 28-day all-cause mortality.

**Figure S2.** ROC curve of NGAL with in 28-day all-cause mortality in SFTS patients.

**Figure S3.** ROC curve of NGAL compared with other risk factors for 28-day all-cause mortality in validation cohort.

**Table S1** Diagnostic performance of NGAL for all-cause mortality.

**Table S2** Baseline characteristics of patients with confirmed SFTS. Baseline characteristics between low NGAL cases and high NGAL cases in the training cohort.

**Table S3** Association of NGAL and mortality outcomes after excluding patients with chronic renal insufficiency of decompensated phase, renal failure phase, and uremia phase.

**Table S4** Association of NGAL and mortality outcomes varied by non-hypertension and hypertension.

**Table S5** Risk factors associated with mortality of SFTS patients by univariate and multivariate cox regression analysis.

**Figure S1.** Subgroup analysis of associations between NGAL and 28-day all-cause mortality. Age, gender, comorbidity (hypertension, diabetes, cancer) were adjusted.

**Figure S2.** ROC curve of NGAL with in 28-day all-cause mortality in SFTS patients.

ROC: receiver operating characteristic.

**Figure S3.** ROC curve of NGAL compared with other risk factors for 28-day all-cause mortality in validation cohort.

ROC: receiver operating characteristic.

**Table S1** Diagnostic performance of NGAL for all-cause mortality.

|  | 14-day all-cause mortality | 28-day all-cause mortality |
| --- | --- | --- |
| optimal cutoff value | 33.915 | 60.597 |
| Area under receiver operating characteristic curve | 0.725 (0.577, 0.872) | 0.752 (0.643, 0.861) |
| Specificity | 0.362 | 0.669 |
| Sensitivity | 1 | 0.75 |
| Accuracy | 0.408 | 0.678 |
| Positive predictive value | 0.109 | 0.211 |
| Negative predictive value | 1 | 0.958 |

**Table S2** Baseline characteristics between low NGAL cases and high NGAL.

| Characteristics | Total (n = 152) | Low NGAL (n = 76) | High NGAL (n = 76) | *p* |
| --- | --- | --- | --- | --- |
| Age, Mean ± SD | 63.0 (54.0, 71.2) | 62.0 (54.0, 71.0) | 64.0 (55.0, 72.0) | 0.409 |
| Gender, n (%) |  |  |  | 0.251 |
| Male | 65 (42.8) | 29 (38.2) | 36 (47.4) |  |
| Female | 87 (57.2) | 47 (61.8) | 40 (52.6) |  |
| Days from symptom onset to admission, Mean ± SD | 6.0 (4.0, 9.0) | 6.0 (4.0, 7.2) | 7.0 (5.0, 9.2) | 0.003 |
| Hospitalization days, Mean ± SD | 9.5 (6.0, 14.0) | 9.0 (6.0, 15.0) | 10.0 (6.8, 13.0) | 0.776 |
| Clinical typing, n (%) |  |  |  | 0.048 |
| 1 | 13 ( 8.6) | 8 (10.5) | 5 (6.6) |  |
| 2 | 69 (45.4) | 41 (53.9) | 28 (36.8) |  |
| 3 | 41 (27.0) | 18 (23.7) | 23 (30.3) |  |
| 4 | 29 (19.1) | 9 (11.8) | 20 (26.3) |  |
| Comorbidity |  |  |  |  |
| Number of comorbidities, n (%) |  |  |  | 0.019 |
| 0 | 67 (44.1) | 41 (53.9) | 26 (34.2) |  |
| 1 | 32 (21.1) | 17 (22.4) | 15 (19.7) |  |
| 2 | 29 (19.1) | 11 (14.5) | 18 (23.7) |  |
| 3 | 13 ( 8.6) | 2 (2.6) | 11 (14.5) |  |
| 4 | 7 ( 4.6) | 4 (5.3) | 3 (3.9) |  |
| 5 | 3 ( 2.0) | 1 (1.3) | 2 (2.6) |  |
| 6 | 1 ( 0.7) | 0 (0) | 1 (1.3) |  |
| Hypertension, n (%) | 45 (29.6) | 19 (25) | 26 (34.2) | 0.214 |
| Diabetes, n (%) | 20 (13.2) | 8 (10.5) | 12 (15.8) | 0.337 |
| Cancer, n (%) | 9 ( 5.9) | 6 (7.9) | 3 (3.9) | 0.494 |
| Clinical features |  |  |  |  |
| Fever, n (%) |  |  |  | 0.373 |
| 37.3~38 | 23 (15.1) | 9 (11.8) | 14 (18.4) |  |
| 38.1~39 | 59 (38.8) | 33 (43.4) | 26 (34.2) |  |
| 39.1~41 | 70 (46.1) | 34 (44.7) | 36 (47.4) |  |
| Digestive symptoms, n (%) | 81 (53.3) | 39 (51.3) | 42 (55.3) | 0.626 |
| Sickness, n (%) | 30 (19.7) | 15 (19.7) | 15 (19.7) | 1 |
| Disturbance of consciousness, n (%) | 43 (28.3) | 17 (22.4) | 26 (34.2) | 0.105 |
| Laboratory tests |  |  |  |  |
| White blood cell (10^9^/L), median (IQR) | 3.0 (1.9, 5.1) | 2.8 (2.1, 4.8) | 3.2 (1.9, 5.1) | 0.975 |
| Red blood cell(10^12^/L), median (IQR) | 4.3 (4.0, 4.7) | 4.3 (4.1, 4.8) | 4.2 (3.8, 4.7) | 0.129 |
| Platelet (10^9^/L), median (IQR) | 65.0 (41.0, 91.5) | 72.5 (52.0, 108.2) | 51.5 (36.8, 74.5) | 0.002 |
| Neutrophils (%), median (IQR) | 64.8 (50.9, 76.6) | 64.3 (49.6, 77.0) | 65.2 (52.3, 76.4) | 0.999 |
| Lymphocytes (%), median (IQR) | 26.1 (16.4, 38.1) | 26.6 (17.7, 37.9) | 24.7 (15.6, 38.1) | 0.636 |
| Hemoglobin (g/L), median (IQR) | 129.0 (117.0, 142.0) | 131.5 (121.0, 144.0) | 128.0 (112.0, 140.2) | 0.136 |
| Alanine transaminase (U/L), median (IQR) | 63.0 (38.8, 117.0) | 56.5 (31.0, 100.0) | 81.0 (46.7, 140.2) | 0.008 |
| Lactate dehydrogenase (U/L), median (IQR) | 649.0 (316.8, 1057.2) | 424.5 (288.8, 825.2) | 815.0 (460.2, 1492.0) | < 0.001 |
| Total bilirubin (µmol/L), median (IQR) | 9.4 (6.2, 13.7) | 9.2 (5.8, 12.6) | 10.1 (6.5, 14.8) | 0.064 |
| Direct bilirubin (µmol/L), median (IQR) | 3.7 (2.3, 6.1) | 3.6 (2.3, 5.2) | 3.9 (2.5, 7.6) | 0.238 |
| Creatine kinase (U/L), median (IQR) | 219.5 (92.0, 693.5) | 189.0 (86.2, 579.8) | 309.5 (98.2, 1054.2) | 0.147 |
| Brain natriuretic peptide (pg/ml), median (IQR) | 7.1 (2.5, 50.6) | 3.4 (2.0, 23.1) | 21.0 (4.7, 129.5) | < 0.001 |
| Triglycerides (mmol/L), median (IQR) | 1.7 (0.9, 2.6) | 1.1 (0.8, 1.9) | 2.0 (1.3, 2.9) | < 0.001 |
| Total cholesterol (mmol/L), median (IQR) | 2.9 (1.7, 3.4) | 2.6 (1.6, 3.4) | 3.0 (2.3, 3.5) | 0.204 |
| High-density lipoprotein cholesterol (mmol/L), median (IQR) | 1.5 (0.9, 191.2) | 14.4 (1.0, 164.6) | 1.1 (0.9, 303.9) | 0.272 |
| Low-density lipoprotein cholesterol (mmol/L), median (IQR) | 0.7 (0.0, 1.5) | 0.1 (0.0, 1.3) | 1.2 (0.2, 1.6) | 0.003 |
| Globulin (g/L), median (IQR) | 28.3 (24.0, 32.9) | 28.4 (23.9, 32.9) | 27.6 (24.1, 32.8) | 0.921 |
| Creatinine (μmol/L), median (IQR) | 69.3 (55.5, 90.8) | 64.8 (52.8, 80.3) | 79.7 (60.4, 104.1) | 0.006 |
| Urine protein, n (%) |  |  |  | 0.191 |
| 0 | 27 (17.8) | 17 (22.4) | 10 (13.2) |  |
| 1 | 33 (21.7) | 20 (26.3) | 13 (17.1) |  |
| 2 | 49 (32.2) | 20 (26.3) | 29 (38.2) |  |
| 3 | 39 (25.7) | 18 (23.7) | 21 (27.6) |  |
| 4 | 4 ( 2.6) | 1 (1.3) | 3 (3.9) |  |
| Blood urea nitrogen (mg/dL), median (IQR) | 5.2 (3.5, 8.2) | 5.1 (3.5, 7.2) | 5.7 (3.6, 8.8) | 0.182 |
| C-reactive protein (mg/L), median (IQR) | 4.9 (2.8, 9.3) | 4.1 (2.3, 7.4) | 6.0 (3.3, 13.7) | 0.017 |
| Prothrombin time (s), median (IQR) | 11.6 (10.8, 12.3) | 11.7 (10.9, 12.1) | 11.5 (10.8, 12.3) | 0.62 |
| International normalized ratio, median (IQR) | 1.0 (0.9, 1.1) | 1.0 (0.9, 1.1) | 1.0 (0.9, 1.1) | 0.362 |
| Amylase (U/L), median (IQR) | 105.0 (69.0, 168.0) | 84.0 (66.5, 121.8) | 132.1 (85.8, 205.2) | < 0.001 |
| Lipase (U/L), median (IQR) | 141.5 (36.0, 353.0) | 70.0 (35.0, 215.7) | 151.5 (47.5, 670.0) | 0.02 |
| Treatment |  |  |  |  |
| Corticosteroid Therapy, n (%) | 41 (27.0) | 25 (32.9) | 16 (21.1) | 0.1 |
| Ribavirin treatment, n (%) | 140 (92.1) | 71 (93.4) | 69 (90.8) | 0.547 |
| Gamma globulin therapy, n (%) | 58 (38.2) | 26 (34.2) | 32 (42.1) | 0.316 |
| Complication |  |  |  |  |
| Myocarditis, n (%) | 28 (18.4) | 16 (21.1) | 12 (15.8) | 0.403 |
| Encephalitis, n (%) | 34 (22.4) | 16 (21.1) | 18 (23.7) | 0.697 |
| Pulmonary infection, n (%) | 49 (32.2) | 24 (31.6) | 25 (32.9) | 0.862 |
| Organ failure, n (%) | 21 (13.8) | 5 (6.6) | 16 (21.1) | 0.01 |
| The number of damaged organs, n (%) |  |  |  | 0.258 |
| 0 | 46 (30.3) | 19 (25) | 27 (35.5) |  |
| 1 | 23 (15.1) | 12 (15.8) | 11 (14.5) |  |
| 2 | 25 (16.4) | 16 (21.1) | 9 (11.8) |  |
| 3 | 30 (19.7) | 18 (23.7) | 12 (15.8) |  |
| 4 | 17 (11.2) | 8 (10.5) | 9 (11.8) |  |
| 5 | 7 ( 4.6) | 1 (1.3) | 6 (7.9) |  |
| 6 | 2 ( 1.3) | 1 (1.3) | 1 (1.3) |  |
| 7 | 2 ( 1.3) | 1 (1.3) | 1 (1.3) |  |
| The number of failed organs, n (%) |  |  |  | 0.038 |
| 0 | 131 (86.2) | 71 (93.4) | 60 (78.9) |  |
| 1 | 14 ( 9.2) | 3 (3.9) | 11 (14.5) |  |
| 2 | 4 ( 2.6) | 1 (1.3) | 3 (3.9) |  |
| 3 | 2 ( 1.3) | 1 (1.3) | 1 (1.3) |  |
| 4 | 1 ( 0.7) | 0 (0) | 1 (1.3) |  |
| 14-day all-cause mortality | 11 ( 7.2) | 3 (3.9) | 8 (10.5) | 0.118 |
| 28-day all-cause mortality | 16 (10.5) | 3 (3.9) | 13 (17.1) | 0.008 |

**Table S3** Association of NGAL and mortality outcomes after excluding patients with chronic renal insufficiency of decompensated phase, renal failure phase, and uremia phase.

|  | Univariate model | | Multivariate model l | | Multivariate model 2 | |
| --- | --- | --- | --- | --- | --- | --- |
|  | HR (95%CI) | *P* | HR (95%CI) | *P* | HR (95%CI) | *P* |
| 14-day all-cause mortality |  |  |  |  |  |  |
| NGAL | 1.016 (1.004~1.027) | 0.006 | 1.013 (1.005~1.021) | 0.002 | 1.013 (1.005~1.021) | 0.002 |
| Low NGAL | Reference |  | Reference |  | Reference |  |
| High NGAL | 2.423 (0.683~8.591) | 0.171 | 1.557 (0.395~6.139) | 0.527 | 1.557 (0.395~6.139) | 0.527 |
| 28-day all-cause mortality |  |  |  |  |  |  |
| NGAL | 1.017 (1.006~1.028) | 0.003 | 1.015 (1.006~1.025) | 0.002 | 1.016 (1.006~1.026) | 0.002 |
| Low NGAL | Reference |  | Reference |  | Reference |  |
| High NGAL | 4.133 (1.313~13.006) | 0.015 | 3.958 (1.258~12.452) | 0.019 | 4.732 (1.422~15.749) | 0.011 |

HR: hazard ratio; CI: confidence interval.

Multivariate model 1: age, gender were adjusted.

Multivariate model 2: age, gender, comorbidity (hypertension, diabetes, cancer) were adjusted.

**Table S4** Association of NGAL and mortality outcomes varied by non-hypertension and hypertension.

|  |  | Univariate model | | Multivariate model l | | Multivariate model 2 | |
| --- | --- | --- | --- | --- | --- | --- | --- |
|  |  | HR (95%*CI*) | *P* | HR (95%*CI*) | *P* | HR (95%*CI*) | *P* |
| 14-day all-cause mortality |  |  |  |  |  |  |  |
| Non-hypertension | Low NGAL | Reference |  | Reference |  | Reference |  |
|  | High NGAL | 5.47 (1.104~27.109) | 0.037 | 4.111 (0.816~20.701) | 0.087 | 4.846 (0.493~47.647) | 0.176 |
| Hypertension | Low NGAL | Reference |  | Reference |  | Reference |  |
|  | High NGAL | 0.558 (0.05~6.161) | 0.634 | 0.583 (0.048~7.16) | 0.673 | 0.535 (0.043~6.723) | 0.628 |
| 28-day all-cause mortality |  |  |  |  |  |  |  |
| Non-hypertension | Low NGAL | Reference |  | Reference |  | Reference |  |
|  | High NGAL | 7.117 (1.511~33.53) | 0.013 | 6.554 (1.379~31.139) | 0.018 | 10.12 (1.204~85.038) | 0.033 |
| Hypertension | Low NGAL | Reference |  | Reference |  | Reference |  |
|  | High NGAL | 1.531 (0.266~8.824) | 0.634 | 1.478 (0.251~8.72) | 0.666 | 1.6 (0.262~9.749) | 0.611 |

HR: hazard ratio; CI: confidence interval.

Multivariate model 1: age, gender were adjusted.

Multivariate model 2: age, gender, comorbidity (hypertension, diabetes, cancer) were adjusted.

**Table S5** Sensitivity analysis additionally adjusting for clinical typing and treatment strategies.

|  | HR (95%*CI*) | *P* |
| --- | --- | --- |
| 28-day all-cause mortality |  |  |
| NGAL | 1.009 (1.003~1.015) | 0.003 |
| Low NGAL | Refernce |  |
| High NGAL | 5.408 (1.241~23.574) | 0.025 |

Age, gender, comorbidity (hypertension, diabetes, cancer), clinical typing, and treatment (corticosteroid therapy, ribavirin treatment, gamma globulin therapy) were adjusted.

**Table S6** Sensitivity analysis additionally adjusting for all recorded complications.

|  | HR (95%CI) | *P* |
| --- | --- | --- |
| 28-day all-cause mortality |  |  |
| NGAL | 1.022 (1.014~1.030) | <0.001 |
| Low NGAL | Refernce |  |
| High NGAL | 7.384 (2.319~23.508) | <0.001 |

Age, gender, comorbidity (hypertension, diabetes, cancer), and complications (myocarditis, encephalitis, pulmonary infection, organ failure, number of damaged organs, number of failed organs) were adjusted.

**Table S7** Risk factors associated with mortality of SFTS patients by univariate and multivariate cox regression analysis.

|  | Univariate model | | Multivariate model l | | Multivariate model 2 | |
| --- | --- | --- | --- | --- | --- | --- |
|  | HR (95%*CI*) | *P* | HR (95%*CI*) | *P* | HR (95%*CI*) | *P* |
| Age | 1.068 (1.019~1.119) | 0.006 | 1.066 (1.017~1.117) | 0.008 | 1.07 (1.017~1.125) | 0.009 |
| NGAL | 1.005 (1.002~1.009) | 0.006 | 1.005 (1.001~1.009) | 0.011 | 1.005 (1.001~1.01) | 0.013 |
| ＜60.597 | Reference |  | Reference |  | Reference |  |
| ≥60.597 | 4.284 (1.378~13.317) | 0.012 | 4.246 (1.365~13.206) | 0.013 | 4.71 (1.466~15.132) | 0.009 |
| PLT | 0.987 (0.972~1.003) | 0.119 |  |  |  |  |
| LDH | 1.001 (1.001~1.001) | < 0.001 | 1.001 (1.001~1.001) | <0.001 | 1.001 (1.001~1.001) | <0.001 |
| TC | 0.575 (0.368~0.899) | 0.015 | 0.575 (0.37~0.893) | 0.014 | 0.575 (0.363~0.911) | 0.019 |
| HDL-C | 1.001 (1.001~1.001) | < 0.001 | 1.001 (1.001~1.001) | <0.001 | 1.001 (1.001~1.001) | <0.001 |
| GLO | 1.061 (0.996~1.129) | 0.067 |  |  |  |  |
| Cr | 1.002 (0.999~1.006) | 0.228 |  |  |  |  |
| BUN | 1.082 (1.003~1.167) | 0.042 |  |  |  |  |
| CRP | 1.004 (0.991~1.017) | 0.581 |  |  |  |  |
| LPS | 1.001 (1.001~1.002) | < 0.001 | 1.001 (1.001~1.002) | <0.001 | 1.001 (1.001~1.002) | <0.001 |

HR: hazard ratio; CI: confidence interval; PLT: platelet; LDH: lactate dehydrogenase; TC: total cholesterol; HDL-C: high-density lipoprotein cholesterol; GLO: globulin; Cr: creatinine; BUN: blood urea nitrogen; CRP: C-reactive protein; LPS: lipase.

Multivariate model 1: age, gender were adjusted.

Multivariate model 2: age, gender, comorbidity (hypertension, diabetes, cancer) were adjusted.
